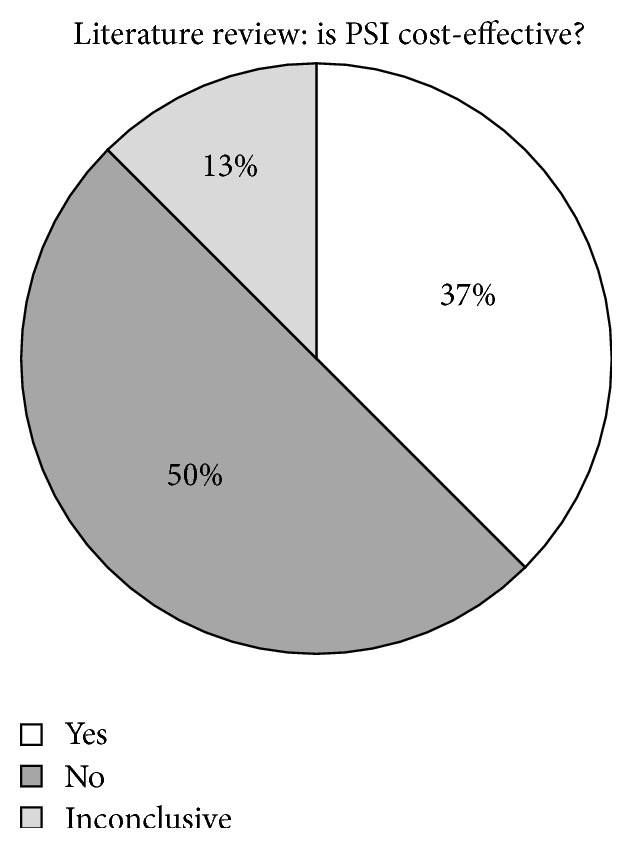# A Systematic Literature Review of Three Modalities in Technologically Assisted TKA

**DOI:** 10.1155/2015/719091

**Published:** 2015-11-18

**Authors:** William A. Leone, Leah C. Elson, Christopher R. Anderson

**Affiliations:** ^1^The Leone Center for Orthopedic Care, Holy Cross Hospital, 4725 N. Federal Highway, Fort Lauderdale, FL 33308, USA; ^2^Department of Bioengineering and Clinical Research, OrthoSensor Inc., 1855 Griffin Road, Suite A-310, Dania Beach, FL 33004, USA

## Abstract

In effort to reduce the revision burden of total knee arthroplasty (TKA), industry emphasis has focused on replacing manual techniques—which are subject to variability—with technological implements. Unfortunately, technological innovation often continues before adequate time for critical evaluation has passed. Therefore, the purpose of this descriptive literature review was to collect a large sample of international data and report on the clinical and economic efficacy of three major types of technologically assisted TKA: navigation, patient-specific instrumentation, and sensorized trials.

## 1. Introduction

Today, digitized tools have become a mainstay in orthopaedic operating rooms around the world. A transition away from manual techniques has been implemented in order to develop new ways to attempt to reduce the proportion of revision total knee arthroplasty (TKA) procedures performed annually. Currently, the United States bears a 2.7-billion-dollar TKA revision burden, a value that has not undergone appreciable decrease in the last decade [1]. Thus, many orthopaedic specialists have looked to intraoperative technological assistance with the hope of reducing the incidence of surgeon-driven error, including component alignment, although it is still unclear if alignment outliers lead to increased revision rates [2, 3].

However, innovation often continues before existing technology has been sufficiently critically evaluated. With more drastic financial restrictions being placed on operating room spending, orthopaedic surgeons are now required to provide excellent results on a budget.

It is integral that both clinical efficacy and cost-effectiveness of these intraoperative technologies be fully understood in order to provide patients with effectual, economically conscious care. Therefore, the purpose of this qualitative analysis of literature was to evaluate clinical and economic efficacy of the three most prominent technologies currently used in TKA: computer navigation, patient-specific instrumentation, and kinetic sensors.

## 2. Methods

A systematic literature review was conducted between September 1, 2014, and September 15, 2014. Using PubMed, combinations of the following keywords were queried: “patient specific instrumentation”, “patient matched instrumentation”, “patient specific cutting blocks”, “computer-assisted surgery”, “computer navigation”, “TKA”, “total knee arthroplasty”, “sensors”, “history”, “cost”, “outcomes”, and “satisfaction,” in the following strings: “patient specific total knee arthroplasty”, “computer navigation total knee arthroplasty,” “sensor total knee arthroplasty”, “patient matched instrumentation TKA,” “patient specific cutting blocks TKA”, “cost sensors TKA”, “cost patient specific instrumentation TKA”, “cost patient matched instrumentation TKA”, “cost patient specific cutting blocks TKA”, “outcomes patient specific instrumentation TKA”, “outcomes patient matched TKA”, “outcomes sensors TKA”, “outcomes computer navigation TKA”, “satisfaction computer navigation TKA”, “satisfaction sensors TKA”, “satisfaction patient specific instrumentation TKA,” “satisfaction patient matched TKA”, “satisfaction patient specific cutting blocks TKA”, “history sensors TKA,” “history computer navigation TKA”, “history patient specific cutting blocks TKA,” and “history patient specific instrumentation.”

**Figure 1:**
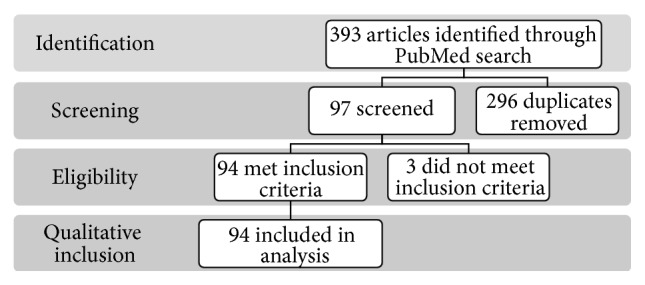


Three hundred and ninety-three publications were collected; 94 were included in final qualitative analysis as per Figure 1. The level of evidence for all harvested publications was as follows: Level 1/Level 2: 52%; Level 3 (all retrospective analyses): 14%; Level 4: 31%; Level 5: 3% (all biomechanical/cadaveric testing). Criteria for inclusion in the analysis were defined only insofar as each piece assessed one of the above listed aspects of patient-specific instrumentation, computer navigation, and/or intraoperative sensors. Literature included in the final evaluation contained background information on each respective technology, clinical outcomes, revision rates, and/or cost analyses. All comparisons were conducted in a strictly qualitative manner, and no attempts were made to conduct interstudy statistical analyses due to the high level of variability in methodology and data collected.

## 3. Results

### 3.1. Computer Navigation

Computer navigation (also called “CAOS” or “computer-assisted orthopaedic surgery”) was developed in order to increase the accuracy of bony resection, while simultaneously decreasing the incidence of positioning outliers. The first documented case of the use of computer navigation, in TKA, was in 1997 [4]. Since then, a variety of navigation systems have been developed.

All systems can be classified into two, broad categories: those which are compatible with specific instrumentation (typically referred to as “closed systems”) or those which can be used regardless of component type or manufacturer (typically referred to as “open systems”) [5]. Navigation is also classified by its method of anatomic mapping, which can be accomplished by CT, fluoroscopy, or imageless means. Each system uses images obtained from the patient (or, in the case of imageless systems, images obtained from a large database), to construct a three-dimensional model of preoperative bony anatomy.

When the patient enters surgery, registration of markers or reflectors is used to define points in space based on the three-dimensional anatomical model. The computer navigation software triangulates the location of each marker and the markers, collectively, provide information regarding location of anatomic landmarks, mechanical axes, component positioning, and center of rotation. Accurate placement of all marker pins in the distal femur and proximal tibia is absolutely crucial for the computer reconstruction of native anatomy.

A survey, conducted by Friederich and Verdonk (*n* = 3,330 surgeons; Swiss Orthopedic Society and European Society of Sports Traumatology Knee Surgery and Arthroscopy) demonstrated that one-third of surgeons use navigation for approximately half of their cases, while one-quarter of surgeons use navigation for over 75% of their cases [6]. Australian Joint Registry data reports a similar proportion of usage: 2015 Hip and Knee Arthroplasty Annual Report indicates 29% of total knee replacements, nationwide, were performed with CAOS. The operative advantages in using navigation to guide TKA are the objective parameters with which bone cuts are made. The accuracy available for obtaining a symmetrical joint gap may also theoretically allow for more balanced soft-tissues [5].

Yet, the results of alignment and positioning have not been consistently reported in literature. While many surgeons agree that navigation allows for more accuracy in component alignment, others argue that no significant difference has been noted when compared with manual medullary techniques [7–35]. For instance, a group of 160 bilateral patients—one knee operated with navigation; one knee operated with manual techniques—showed that there was no significant difference with respect to alignment [26]. Yet, a meta-analysis, conducted by Hetaimish et al. contends that an evaluation of 23 publications agree that positioning outliers are greatly reduced with the use of navigation [36].

**Figure 2:**
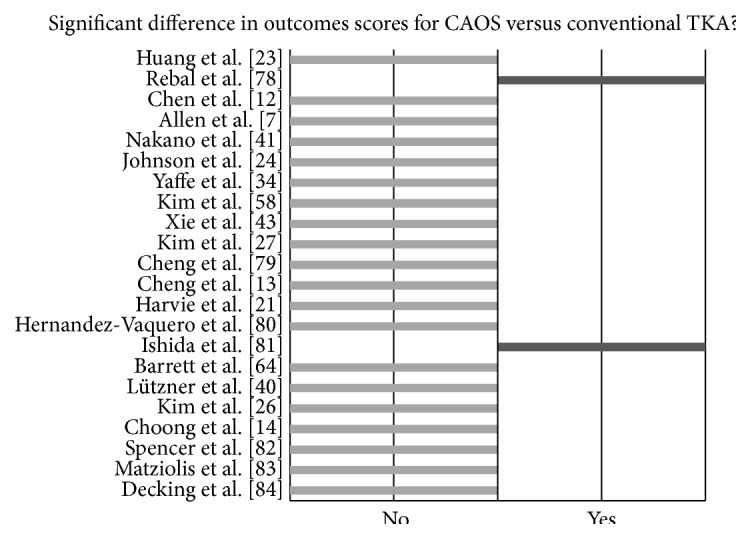


However, even with confirmed alignment accuracy, the clinical outcomes of patients present another aspect of navigation that is widely debated [7, 9, 13, 18, 20, 24, 31, 37–44]. Many surgeons have made note of marked improvement in groups of navigated patients. A survivorship analysis, performed by Hakki et al., showed a lack of revision surgery required in navigated TKAs, at 5 years, while the nonnavigated group showed a 2.8% revision rate at the same time interval (*n* = 100) [39]. Another publication, using data from the Australian registry, shows reduced revision rates, in patients younger than 65 years, who have undergone navigated TKA [45]. Yet, other surgeons have reported no major difference in outcomes measures when compared with manual TKA (Figure 2). A recent study from 2014 showed no difference in navigated versus nonnavigated survivorship duration, KSS outcomes measures, and HSS outcomes measures at 5 years postoperatively [15].

Beyond an overall divided opinion on the effectiveness of navigation, the price point of navigation is also quite high. The price of a typical system is calculated in a piecemeal fashion: cost of the computer, cost of the software, and cost of an annual service contract from the manufacturer. In total, the dues of navigation equipment, per year, can reach upwards of $45,000 dollars [46–48]. Many surgeons have attempted to analyze the cost-effectiveness by assessing the frequency of use. Three particular evaluations have noted that navigation systems have the potential to be cost-effective, but only in high-volume medical centers [47, 49, 50]. Still, a paper by Gøthesen et al. contends that the purchase of a navigation package is* only* cost-effective at high-volume centers,* if* it proves to significantly decrease revision rates [46]. On a cost-per-case basis, navigation has been shown to contribute to an additional $1,500 per procedure [47].

### 3.2. Patient-Specific Instrumentation

Due to the impact of high costs associated with navigation and the complex protocol associated with registering markers, patient-specific instrumentation (PSI) was developed to increase cutting accuracy, decrease the amount of surgical trays required, and simplify cutting procedures.

This intraoperative technology was introduced in the first decade of the 21st century [51, 52] and is now available through six separate manufacturers [52]. PSI usage begins with preoperative imaging, which can combine CT, MRI, and/or a standing anteroposterior radiograph. A three-dimensional model is constructed from the amalgam of imaging techniques and a positioning algorithm (specific to each manufacturer) is applied to determine the correct positioning for the tibial and femoral components. This preoperative plan is then sent to the surgeon for approval. Once the surgeon has confirmed the preoperative plan, the PSI is rendered into the physical cutting jig system. Intraoperatively, the femoral guide is clicked into place and is used to determine sizing, level of resection, rotation, and anteroposterior positioning. Similarly, the tibial guide assists in the determination of tibial alignment, rotation, level of resection, and slope. The multifaceted capability of the PSI system eliminates several steps normally attributed to femoral and tibial preparation, theoretically increasing operating room efficiency [53].

Several studies have confirmed that the truncation of steps associated with using PSI does decrease operative time [54–59]. One particular study noted an average 20-minute decrease in operating room time, when compared with manual TKA [60].

In addition to the general consensus on time saving, many authors also agree that alignment with PSI is often inaccurate or exhibits a larger proportion of outliers than conventional TKA [61, 62]. One particular evaluation noted no difference between PSI and conventional method alignment in femoral coronal and femoral axial planes but a marked increase in PSI outliers in both the coronal and sagittal tibial planes (*n* = 128) [63]. Another study observed a 21% increase in hip-knee-ankle angle outliers with the use of PSI, when compared with conventional methods [61]. A majority of studies have indicated that PSI is either as accurate as or less accurate than conventional TKA or navigation systems [50, 58, 64–73].

A consensus on inaccuracy might beg the question: why? If the cutting jigs are custom-rendered to fit each patient's specific bony morphology, where does the incidence of inaccuracy originate from? Several surgeons sought to answer this question, and many of them observed that the preoperative plan did not match the final, physical rendering used in the operating room [62, 74–76]. One particular study, conducted by Scholes et al., noted that 27% of the PSI cutting jigs, received in surgery, induced coronal error in excess of 3° [76]. Another study also showed that 77% of femurs, and 54% of tibias, required intraoperative resizing due to lack of fit [62]. The authors of these studies came to similar conclusions: because there are several steps associated with creating the jigs, there is opportunity for error. Due to the process associated with rendering PSI (imaging, model creation, planning, and manufacturing), involving the surgeon and manufacturing engineers, small errors may culminate in ineffective cutting jigs. All authors have advised that great care be taken on part of the surgeon when approving the preoperative plans and avoiding using PSI in any procedure without prior approval of jig schematics [62, 74–76].

Evidence surrounding the clinical outcomes of PSI is sparse. However, much of it draws similar conclusions. One study noted that there seemed to be improved postoperative kinematics, when compared with manual TKA, but there was no difference in KSS, quality of life, KOOS, or SF-12 scores [77]. Another study indicated that the tibial slope exhibited more precision in PSI patients, but their blood loss, pain, satisfaction, and functional outcomes were comparable to manual TKA patients [73]. The only study from this evaluation that noted a higher postoperative functional improvement, in PSI patients, also indicated that the preoperative scores were higher to begin with, thus being inconclusive evidence for improvement [34].

Cost-effectiveness for PSI can be evaluated in several ways; PSI may reduce operative time, requires less equipment, and is less expensive instrumentally. However, much of the literature is also divided on the validity of these claims (Figure 3). Several studies indicate that PSI* is* cost-effective [57, 59, 60, 85, 86]. One study, by Lionberger et al., showed that the time saved in using PSI allows for an increased volume of procedures, citing that 3 PSI procedures can be completed for every 2 navigated procedures, resulting in a 1.45x increased profit [85]. Several more studies observe that PSI is* not* cost-effective [75, 87, 88]. One study contends that while there were fewer trays required for the procedure, the inaccuracy of the cutting jigs required more work intraoperatively, thereby negatively impacting the operating room efficiency [75]. On a cost-per-case basis, PSI has been shown to contribute upwards of $1,000 per procedure in vendor charges to the hospital (cost of fabrication of cutting blocks)* and* includes up to $1,000 dollars in additional charges for imaging [58, 88, 89].

### 3.3. Intraoperative Sensors

Correct alignment in TKA only represents a portion of operative factors that contribute to component survivorship and patient satisfaction. In effort to quantify soft-tissue balance, intraoperative kinetic sensors have been developed to dynamically guide surgeons through ligament release. This technology is the most recent innovation in TKA and has been introduced within the last 10 years [90].

Kinetic sensor systems are composed of a sensor (a small housing containing a microprocessing unit and medial-lateral force plates) and a software system which provides the surgeon with dynamic, visual output of force vectors and tibiofemoral contact point location. The sensor fits into the tibial baseplate and is used to track loading values as the surgeon guides the knee joint through a range of motion. Using the visual output, the surgeon can selectively resect more bone or release soft-tissues in order to quantifiably balance the knee. Additionally, using the tibiofemoral contact point positions, in both the medial and lateral compartments, relative to one another, measurements of tibial tray rotation can be captured and corrected [91, 92]. No additional time is necessary in using the sensor, and it has not been reported to disrupt surgical workflow [91, 92].

Studies have confirmed that kinetic sensors are sensitive enough to measure force differentials as small as 1 lb. per square inch and with error margins within 1.5% [93]. Furthermore, these same studies have also confirmed that subtle imbalance, as detected by the sensor, can be seen clinically. A study by Wasielewski et al. used fluoroscopic imaging to demonstrate that imbalance detected by the sensor manifests as unfavorable kinematics during gait [94]. Another study showed that initial placement of the sensor displayed imbalance in all knees tested and that the sensor substantially reduced imbalance before closure [95].

While only recent articles exist regarding clinical efficacy of such a new technology, the research available suggests favorable clinical outcomes associated with using kinetic sensors to achieve balance, at both short- and long-term intervals. One multicentric study, by Gustke et al., showed postoperative improvement in WOMAC, KSS, and activity level scores in patients balanced with sensor assistance [96]. Another study showed further significant improvement, for the same group of multicenter patients at 1 year and for patient satisfaction [89, 97]. There was also an observed trend towards clinically relevant weight loss (weight loss > 6 lbs.) in a group of patients balanced with sensor assistance when compared with literature-reported values [98].

One particular study evaluating a specific sensor type claims that the sensor is both disposable and priced under $1,000 per case and currently available with several total knee systems, although this pricing schema has not been confirmed elsewhere [99].

When comparing outcomes qualitatively, the Knee Society Score (KSS) was the most frequently reported metric for all three technologies and gave the authors a consistent point of comparison, with a follow-up of one or two years postoperatively. Kinetic sensors appeared to offer the highest increase in KSS scores and lowest cost-per-case. PSI data showed the second-highest increase in KSS scores but the most costly increase in excess procedural fees. CAOS was associated with the least highest increase in KSS and the second-highest cost-per-case [12, 19, 27, 46–49, 52, 54, 60, 85, 88, 99–101]. However, high quality, long-term, randomized trials for kinetic sensors have* not* yet been published and will be essential for a more thorough understanding of any clinical efficacy. Furthermore, quality adjusted life years analyses will also be required to understand the cost-effectiveness, as a function of health, for these devices.

## 4. Discussion

Advances in electrical engineering have made it possible for surgeons to use technology to reduce the subjectivity associated with TKA procedures. Currently, technological innovation is being driven with the hope of dramatically reducing the crippling 2.7-billion-dollar revision burden in the United States [1]. However, many of these novel devices are developed before a thorough understanding of the clinical and economic implications of prior devices is fully made. Therefore, it is imperative that overall efficacy of these technologies is explored and considered before implementing them into a clinical setting.

Three prominent technologies are used in modern orthopaedic operating rooms, including computer navigation, patient-specific instrumentation, and kinetic sensors. Navigation has been on the market for the longest duration of time, followed by patient-specific instrumentation and finally—the newest device—kinetic sensors. Each device has been developed with the goal of improving the accuracy of operative procedures and increasing patient satisfaction.

Navigation was designed to reduce alignment and component positioning outliers. While many surgeons have vouched for its precision [20, 25, 29, 36, 79], many more have argued that its results are no better than that achieved by manual techniques [7, 12, 102, 103]. Further divided is the topic of clinical outcomes. Studies have shown that clinical outcomes have improved in navigated TKA patients [14, 30, 39, 42], but an abundance of research suggests that this is not the case [11, 13, 15, 22, 24, 26, 38, 40, 41, 43, 44]. In consideration of the expense of this technology [47, 48], coupled with inconclusive results, navigation does not, at this time, seem to fit the schema for significantly reducing the rate of revision and operative cost.

Patient-specific instrumentation was designed to reduce the expense of navigation systems, simplify computer-assisted methods, and improve functional outcomes [53]. However, a majority of research has suggested that PSI is either no better, or even worse, at alignment accuracy than manual techniques [50, 58, 61, 63–73, 104]. This inability to consistently reduce positioning outliers may be a product of the convoluted system with which each PSI is rendered [74–76, 104]. Because of this, many surgeons have reported that PSI actually increases operative time by disrupting workflow [75]. Also, very few publications have been able to attest to any significant increase in functional outcomes scores of PSI patients, over the scores of navigation or manual TKA [34, 73]. Perhaps with further refining of the PSI rendering process, accuracy can be improved. However, PSI currently does not prove to reduce TKA complications or decrease operating room costs [75, 87, 88].

Finally, kinetic sensor technology has been engineered to quantify soft-tissue balance, improve rotational alignment, and decrease the risk of postoperative complications. The margin of error for detecting loads has been shown to be low [93]; the sensors may be able to measure subtle imbalance that leads to altered gait kinematics [94] and has shown improvement in several patient-reported outcomes measures in balanced patients [55, 96, 99, 100]. This technology may prove to be promising in that it does not add appreciable time to surgical workflow and may also be cost-effective [91, 92, 99].

There were limitations to this literature review. (1) There was no quantitative analysis performed. The variability and scope of topics and procedures discussed would make confounding standardization difficult, statistically. Thus, in order to keep the evaluation simple, and mitigate the risk of an improper application of statistics, analyses were limited to qualitative modalities. (2) Robotic total knee arthroplasty is not reviewed. Although this is an additional technological innovation it is not available for all major knee systems. As such, the authors sought to compare modalities that could be applied to the largest base of component types for applicability to the readers. (3) There is limited economic and clinical data for the sensor modality. Because this is the most recent implement for total knee arthroplasty, Level I research evaluations have yet to be published, and the longest published follow-up interval is at 1 year. However, the authors thought it important to include sensorized data because it is the only modality that assists the surgeon in evaluation of soft-tissue, exclusively, is novel in its disposability, and thus represents a stark contrast to PSI or CAOS for comparison purposes.

If innovation is directed responsibly, both clinical efficacy and cost-effectiveness are attainable for the future of TKA. This review shows some technologies may not yield a clinical or time-saving payoff for the patient and hospital. While kinetic sensor devices seem to be the most promising modality, much more research will be necessary to confirm its advantages over time. But, great care must be taken when adopting any novel technology; “new” does not always mean “improved.”

## Figures and Tables

**Figure 3:**